# Supplementary material for: A feature-based approach for atlas selection in automatic pelvic segmentation
Source: PLoS One. 2025 Jan 30;20(1):e0317801. doi: 10.1371/journal.pone.0317801 (PMC11781710; doi:10.1371/journal.pone.0317801)
Supplement: Attachment — Submitted filename: PONE-D-23-24775_reviewer.docx [file pone.0317801.s001.docx]

Manuscript #PONE-D-23-24775

“A feature-based approach for atlas selection in automatic pelvic segmentation”

Peer-reviewed by Dr. Johann Hêches

Overall, the proposed manuscript is well written and of interest.

Any research that leads to improving accuracy and computational cost of automatic image segmentation methods is very welcomed as manual segmentation is exhausting.

Please find my comments underneath, may they hopefully help you to improve the quality of this manuscript.

A/

“These routines compute the MI between two images after rigid registration using

the method of Mattes[19]. Mutual information (MI)”

Consider defining the “mutual information (MI)” abbreviation at its first occurrence in the text.

B/

“𝐷𝑆𝐶(𝐴, 𝐵) = 2|𝐴 ∩ 𝐵|/|𝐴| + |𝐵|”

A parenthesis is missing in this formula, |B| should be part of the fraction denominator.

C/

“We also suggested a feature-based atlas selection approach (MAS-FASA) that atlas with the closest feature space distance is selected as the candidate for MAS segmentation without including any additional atlases, thus reducing the atlas search time”

Either some punctuation might be missing or the sentence should be rewritten. The sentence is understandable but it was very confusing to read at first.

D/

Conclusion is missing for the manuscript ?
