# Supplementary material for: A feature-based approach for atlas selection in automatic pelvic segmentation
Source: PLoS One. 2025 Jan 30;20(1):e0317801. doi: 10.1371/journal.pone.0317801 (PMC11781710; doi:10.1371/journal.pone.0317801)
Supplement: Attachment — Submitted filename: Response to Reviewers#2.docx [file pone.0317801.s002.docx]

Manuscript #PONE-D-23-24775

“A feature-based approach for atlas selection in automatic pelvic segmentation”

Peer-reviewed by Dr. Johann Hêches

Overall, the proposed manuscript is well written and of interest.

Any research that leads to improving accuracy and computational cost of automatic image segmentation methods is very welcomed as manual segmentation is exhausting.

Please find my comments underneath, may they hopefully help you to improve the quality of this manuscript.

A/

“These routines compute the MI between two images after rigid registration using the method of Mattes[19]. Mutual information (MI)”

Consider defining the “mutual information (MI)” abbreviation at its first occurrence in the text.

Thank you for pointing out the shortcomings of the article. The term 'Mutual information' has been abbreviated and defined upon its first occurrence. These similarity metric includes similarity index[6], the sum of squared difference of image intensity[7], correlation coefficient [8], and mutual information (MI) [9].”

B/

“𝐷𝑆𝐶(𝐴, 𝐵) = 2|𝐴 ∩ 𝐵|/|𝐴| + |𝐵|”

A parenthesis is missing in this formula, |B| should be part of the fraction denominator.

Thank you for identifying the flaws in the article. They have been addressed as: $DSC(A,B)=2|A\cap B|/(\left| A \right|+\left| B \right|)$

C/

“We also suggested a feature-based atlas selection approach (MAS-FASA) that atlas with the closest feature space distance is selected as the candidate for MAS segmentation without including any additional atlases, thus reducing the atlas search time”

Either some punctuation might be missing or the sentence should be rewritten. The sentence is understandable but it was very confusing to read at first.

Thank you for pointing out the flaws in the article. The following modifications have been made:

We also suggested a feature-based atlas selection approach (MAS-FASA), where the atlas with the closest feature space distance is selected as the candidate for MAS segmentation. This method eliminates the need for additional atlases, resulting in reduced atlas search time.

D/

Conclusion is missing for the manuscript ?

Thank you for pointing out the shortcomings of the article. A conclusion section has been added with the following content:

Conclusion

This study proposed a subgrouping method for MAS, which aided in searching for the most fitting atlases based on multiple features. The proposed MAS-SAGA revealed improvements in segmentation performance compared to conventional MAS (cMAS) approach, with the DSC for the bladder and rectum escalating from 0.69±0.15 to 0.83±0.09 and from 0.56±0.16 to 0.70±0.07, respectively. Furthermore, we conducted a comparative analysis between two atlas selection methods, similarity and volume features, and found that the consistency in candidate atlas selection between the two methods was only 4%, indicating significant disagreement between the two approaches. Hence, the integration of volume features into atlas search contributes to enhancing the segmentation performance of MAS.
