# Supplementary material for: A feature-based approach for atlas selection in automatic pelvic segmentation
Source: PLoS One. 2025 Jan 30;20(1):e0317801. doi: 10.1371/journal.pone.0317801 (PMC11781710; doi:10.1371/journal.pone.0317801)
Supplement: Attachment — Submitted filename: Response to Reviewers.docx [file pone.0317801.s003.docx]

**Overall this manuscript about multi atlas segmentation using subset atlas group approach is well-written. The stated aim of comparing cMAS with new technique is clear. More guidance should be provided to the readers to make the results more accessible.
The idea is good; however, I have some comments about improving the current version.

In the Introduction section, the advantage of MAS over DL has been discussed multiple times. What is the rationale behind choosing image similarity and volumetric features?**

Thank you for your suggestions. We have included the following content in the introduction section: Our study is based on the following hypothesis that similarity-based atlas selection methods tend to search for atlases with high overall image similarity. However, when dealing with large-scale deformation, the accuracy of deformation modeling is often compromised due to the involvement of significant nonlinear deformations, geometric complexities, and the impact of intricate boundary conditions and constraints. It may lead to instability in segmentation results, making further refinement challenging.

**Can you explain if the atlases selected are the representative sample of population? (That represents better anatomical variability?)**

Thank you for your suggestions. We have included the following content in the Result section: The 100 atlas samples that were selected are representative, with bladder volumes ranging from 70.89cc to 437.09cc and rectal volumes ranging from 21.3cc to 115.04cc.

Their volume distributions are shown in the figure. The data were randomly selected from the patient database, and the volume distribution is typical. We believe this approach enhances the robustness of the model.

**Do you think different warping techniques and parameters would affect the fusion?**

We believe that different warping techniques can affect the fusion. By setting specific Young's Modulus values for each type of organ, the resulting DVF can be adjusted. Some algorithms achieve deformation by controlling the displacement of feature points, while others use finite element methods. Different algorithms and parameter settings vary in handling different types of organs and boundaries, leading to different final results.

According to the reviewers' comments, we have made the corresponding revisions in the Discussion section.

**Can you explain the robustness of the technique for example, atlas with different scanner characteristics and sample demographics?**

The data we used came from two different types of scanners, the GE LightSpeed CT scanner and the Brilliance CT Big Bore scanner. We did not observe any influence of different scanners on deformation results, nor did we find any relevant reports. It is clear that the demographics of the samples can affect deformation results, so we also considered their representativeness when selecting atlases.

According to the reviewers' comments, we have added the following content to the Method section: To the best of our knowledge, there is no evidence indicating that different CT scanners have a significant impact on atlas segmentation results. Therefore, we did not follow any specific inclusion or exclusion criteria when selecting images.

**Please explain the cross validation results (training and test set)?**

Thank you for the reviewers' suggestions. Based on these insights, we have supplemented the content in the Results section.

We randomly selected 70 out of 100 images as the atlas set and used the remaining 30 for validation. The training set was randomly selected three times with the following group results: (21, 19, 17, and 13), (20, 14, 15, and 21), and (17, 16, 18, and 19). Subsequently, contour comparison was conducted on the 30 image sets, calculating DSC and 95HD. Unlike deep learning cross-validation methods which evaluate convergence with loss functions on test sets, MAS methods do not exhibit overfitting during training. We tested cMAS, MAS-SAGA, MAS-FASA, and MAS-SIM, with results shown in Table 1. Cross-validation results indicate that incorporating volume features as classification criteria leads MAS-SAGA and MAS-FASA to achieve superior DSC and 95HD scores with lower variance compared to cMAS.
